# *TBX2* subfamily suppression in lung cancer pathogenesis: a high-potential marker for early detection

**DOI:** 10.18632/oncotarget.19938

**Published:** 2017-08-04

**Authors:** Athar A. Khalil, Smruthy Sivakumar, Frances Anthony San Lucas, Tina McDowell, Wenhua Lang, Kazuhiro Tabata, Junya Fujimoto, Yasushi Yatabe, Avrum Spira, Paul Scheet, Georges Nemer, Humam Kadara

**Affiliations:** ^1^ Department of Biochemistry and Molecular Genetics, Faculty of Medicine, American University of Beirut, Beirut, Lebanon; ^2^ Department of Epidemiology, University of Texas MD Anderson Cancer Center, Houston, Texas, USA; ^3^ University of Texas MD Anderson Cancer Center UTHealth Graduate School of Biomedical Sciences, Houston, Texas, USA; ^4^ Department of Translational Molecular Pathology, University of Texas MD Anderson Cancer Center, Houston, Texas, USA; ^5^ Graduate School of Biomedical Science, Nagasaki University, Nagasaki, Japan; ^6^ Department of Pathology, Aichi Cancer Center, Nagoya, Japan; ^7^ Section of Computational Biomedicine, School of Medicine, Boston University, Boston, Massachusetts, USA

**Keywords:** NSCLC, smoking, preneoplasia, airway field of injury, early detection

## Abstract

The *TBX2* subfamily (*TBXs* 2, 3, 4 and 5) transactivates or represses genes involved in lung organogenesis. Yet *TBX2* subfamily expression in pathogenesis of non-small cell lung cancer (NSCLC), the most common lung malignancy, remains elusive. We sought to probe the expression profile of the *TBX2* subfamily in early phases of NSCLC. Expression of *TBX2* subfamily was analyzed in datasets of pan-normal specimens as well as NSCLCs and normal lung tissues. *TBX2* subfamily expression in matched normal lungs, premalignant hyperplasias and NSCLCs was profiled by transcriptome sequencing. *TBX2* subfamily expression was evaluated in the cancerization field consisting of matched NSCLCs and adjacent cytologically-normal airways relative to distant normal lungs and in a dataset of normal bronchial samples from smokers with indeterminate nodules suspicious for malignancy. Statistical analysis was performed using R. *TBX2* subfamily expression was markedly elevated in normal lungs relative to other organ-specific normal tissues. Expression of the *TBXs* was significantly suppressed in NSCLCs relative to normal lungs (*P* < 10^−9^). *TBX2* subfamily was significantly progressively decreased across premalignant lesions and NSCLCs relative to normal lungs (*P* < 10^−4^). The subfamily was significantly suppressed in NSCLCs and adjacent normal-appearing airways relative to distant normal lung tissues (*P* < 10^−15^). Further, suppressed *TBX2* subfamily expression in normal bronchi was associated with lung cancer status (*P* < 10^−5^) in smokers. Our findings suggest that the *TBX2* subfamily is notably suppressed in human NSCLC pathogenesis and may serve as a high-potential biomarker for early lung cancer detection in high-risk smokers.

## INTRODUCTION

Lung cancer is the leading cause of cancer deaths in the United States and worldwide [1]. The most commonly diagnosed type of lung cancer (about 85%) is non-small-cell lung cancer (NSCLC), which comprises two main histological subtypes, adenocarcinoma (LUAD) and squamous cell carcinoma (LUSC) [2]. The high mortality rates associated with NSCLC are largely due to late diagnosis [3]. Early detection strategies for NSCLC are still very limited [4, 5]. While screening by low-dose computed tomography was shown to reduce lung cancer mortality, this approach exhibits substantial false positive rates warranting new or complimentary strategies [6]. Limitations in achieving these advances are, in part, due to our lagging knowledge of early (e.g., premalignant) molecular alterations in NSCLC pathogenesis. There are few molecular alterations that have been described in premalignant lung lesions; these include mutations in the *KRAS* oncogene and loss of heterozygosity (LOH) in chromosomal regions 3p and 9p [7]. Earlier studies have pinpointed molecular changes in cytologically-normal airway epithelium that are shared with the lung tumor itself, a phenomenon referred to as “airway field of injury” [8–10]. Since they manifest in normal-appearing epithelium, airway field of injury changes are suggested to be highly pertinent to the pathobiology and early clinical management of NSCLC [10].

T-box (*TBX*) genes encode a family of evolutionarily conserved transcription factors implicated throughout organogenesis and development [11, 12]. *TBX* genes play essential roles in differentiation, proliferation and tissue integrity [13, 14]. Mutations in these factors are causally linked to inherited genetic disorders in humans [13, 14]. In mice, members of the *Tbx2* subfamily (*Tbx* 2, 3, 4 and 5) are expressed in the developing lungs (lung buds and trachea) [15, 16]. *Tbx2* was shown to be required in maintaining normal proliferation of lung mesenchyme in murine lungs with depletion of the gene [17]. *Tbx2*-deficient embryonic lungs exhibit reduced branching, and proper lung branching is largely mediated by *Tbx2* and *Tbx3* [18]. The other cognate gene pair, *Tbx4* and *Tbx5*, also play essential roles during mammalian development. In mice, depletion of *Tbx4* or *Tbx5* hampers bronchial differentiation in *ex-vivo* lung cultures [19]. Moreover, targeted inducible inactivation of *Tbx5*, but not of *Tbx4*, was demonstrated to inhibit lung bud and tracheal formation [19].

While *Tbx2* subfamily plays crucial roles in normal mouse lung development, the profile of these transcription factors in human NSCLC is unknown. Here, we sought to survey *TBX2* subfamily expression in different phases of human NSCLC pathogenesis. We report that all four members of the *TBX2* subfamily are suppressed in NSCLCs and premalignant precursor lesions as well as in the cancerization field in the normal-appearing airway adjacent to tumors. Lastly, we demonstrate that suppressed expression of the *TBX2* subfamily in normal airways is indicative of lung cancer status in smokers suggesting that this subfamily can serve as a high-potential biomarker for early lung cancer detection.

## RESULTS

### Preferential expression of *TBX2* subfamily in normal lung tissues

**Figure 1:**
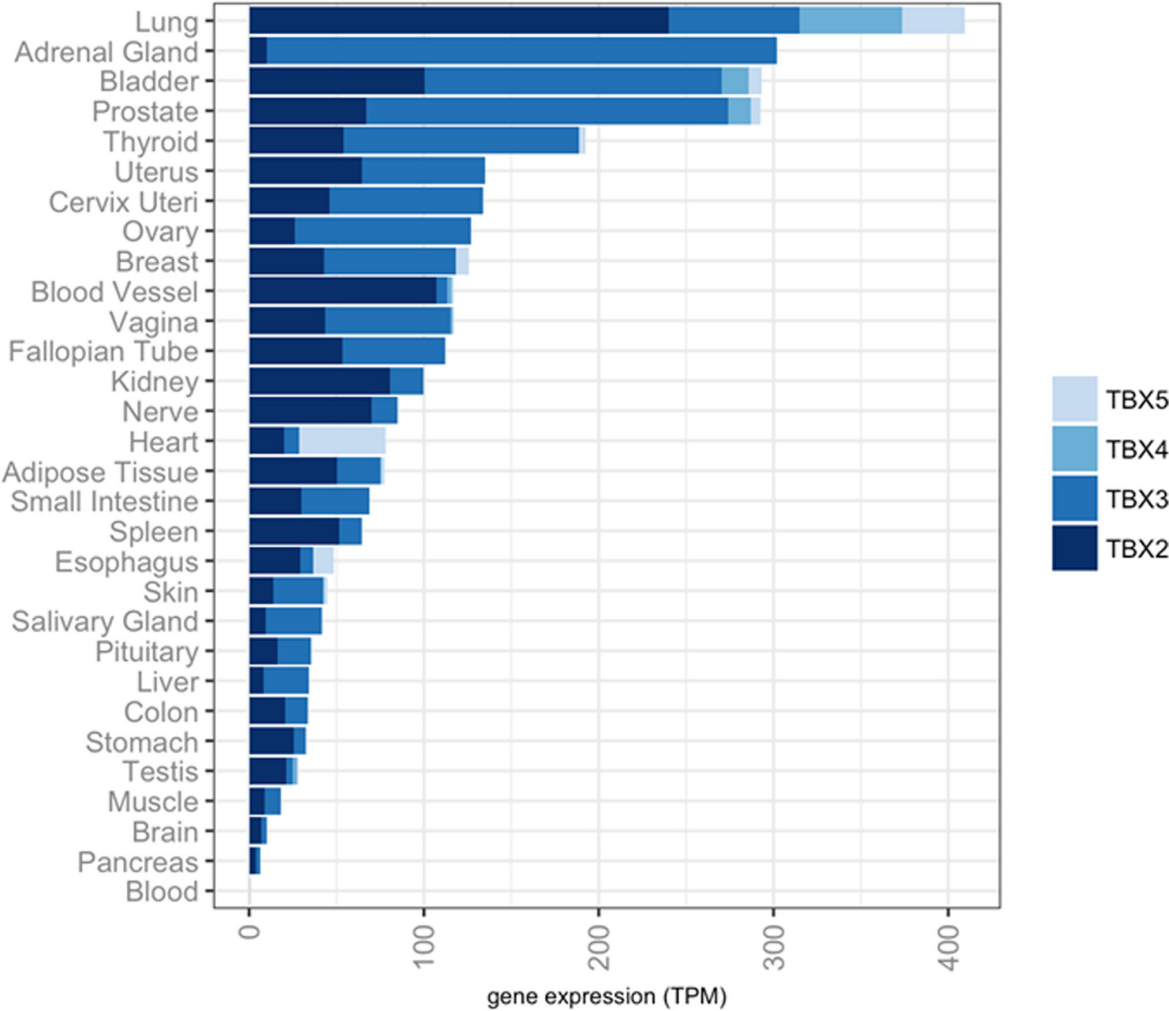
Preferential expression of the *TBX2* subfamily in human normal lung Expression levels of *TBX2*, *TBX3*, *TBX4* and *TBX5* mRNAs were analyzed in > 7,000 pan-normal specimens using the Genotype Tissue Expression Project (GTEx) and plotted in R. RNA-sequencing based gene expression values are denoted as transcripts per million (TPM).

The *TBX2* subfamily was shown to be highly expressed in normal mouse lung [18–21] yet, its expression and function in normal human lung tissues is poorly understood. Here, we sought to survey the expression of this subfamily in human normal tissues by *in silico* analysis of publicly available expression datasets within the genotype tissue expression (GTEx) project constituting > 7,000 human pan-normal tissues [22]. Our analysis demonstrated that all four members were markedly abundantly expressed in normal lung compared with other organ-specific normal specimens (Figure 1). These data point to human lung lineage-specific expression patterns for the *TBX2* subfamily of transcription factors.

### Suppressed expression of the *TBX2* subfamily in NSCLCs relative to normal lung tissues

**Figure 2:**
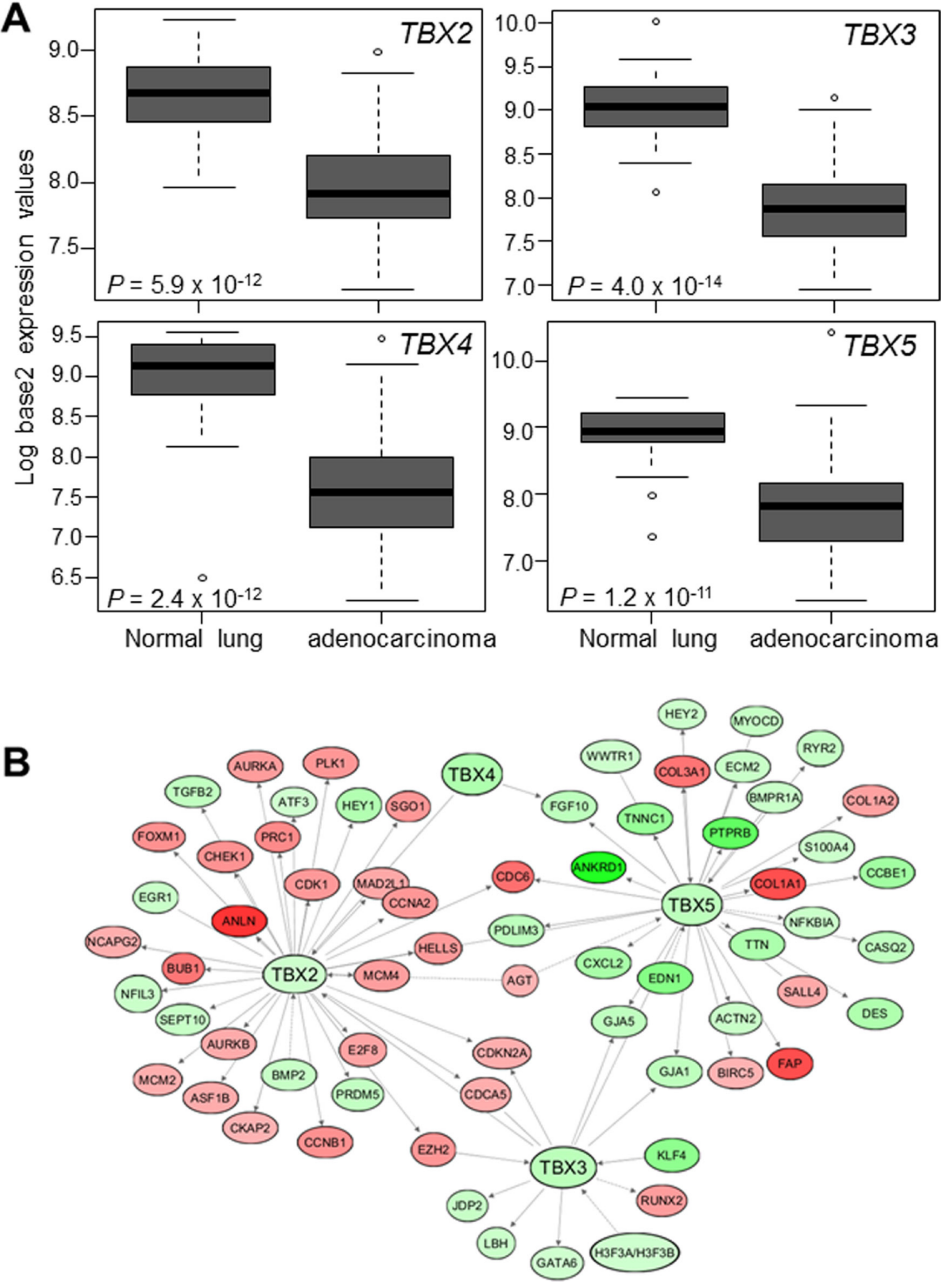
Suppressed expression of the *TBX2* subfamily in human NSCLC (**A**) Expression levels of *TBX2* subfamily were probed in a normalized expression dataset of 80 LUADs and 30 normal lung tissues we previously reported [23]. Expression differences were statistically analyzed using Wilcoxon rank sum tests. Boxes represent 25%–75% ranges. Solid horizontal lines represent median values. (**B**) Known downstream *TBX2* subfamily target genes that are differentially expressed between LUADs and normal lung tissues (FDR < 0.05, fold-change ≥ 2), along with the subfamily itself, were organized into gene networks using Ingenuity Pathways Analysis (IPA) (red; up-regulated in tumors; green, down-regulated).

We then statistically compared and contrasted expression of the four *TBX2* subfamily members in an expression dataset of NSCLCs and normal lung tissues we previously reported [23]. We found that all four members were significantly and notably decreased in LUADs relative to normal lung tissues (Figure 2A; all *P* < 10^−10^). Expression of the *TBX2* subfamily significantly distinguished LUADs from normal lung tissues (Supplementary Figure 1, *P* < 0.001 of the Fisher's exact test). Of note, expression of all four *TBX2* subfamily members in LUADs and normal lung tissues was statistically highly positively correlated (all *P* < 0.001; Supplementary Figure 2). Further, we confirmed the suppressed expression of the *TBX2* subfamily in NSCLCs by analysis of additional publicly available expression datasets [24–26] (all *P* < 10^−9^, Supplementary Figure 3). We then performed gene-gene interaction network analysis to identify topologically-organized genes downstream of *TBX2*, *TBX3*, *TBX4* and *TBX5* and that are differentially modulated in LUADs relative to normal lung tissues. This analysis underscored canonical tumor promoting genes (e.g. cell cycle promoting genes such as *CDK1* and *BUB1*) that were up-regulated in LUADs relative to normal lung tissues and concomitantly downstream of down-regulated *TBX2* with the same gene-network (Figure 2B). These results point to commonly occurring suppressed expression of the *TBX2* subfamily in human NSCLC.

### Down-regulation of the *TBX2* subfamily in premalignant lung lesions

**Figure 3:**
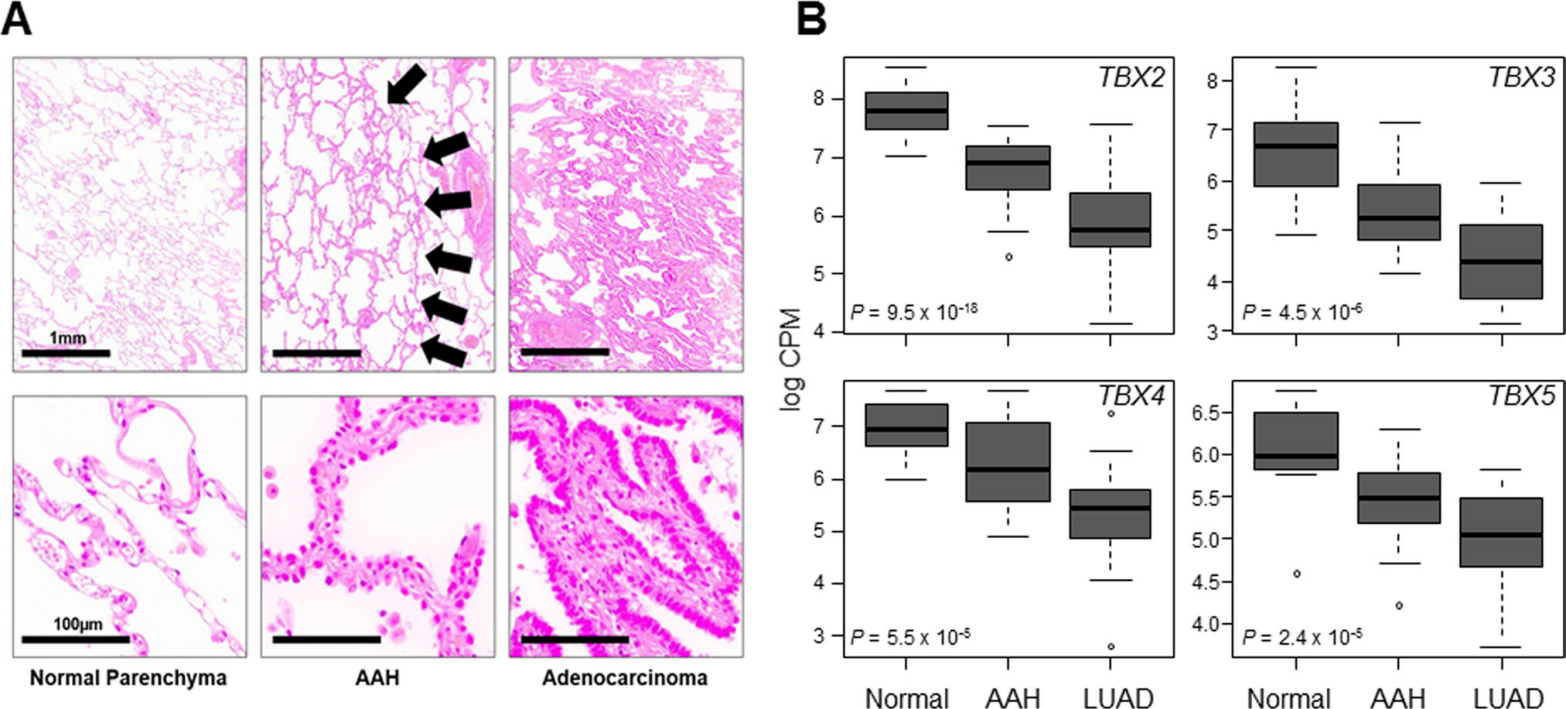
Progressive down-regulation of *TBX2* subfamily in premalignant and malignant lung tissues FFPE specimens from normal lung tissues, AAHs and LUADs (from 17 patients) were analyzed by transcriptome sequencing using the Ion Torrent Proton platform (see Materials and Methods). (**A**) Representative photomicrographs of a matched set of tissues. Arrows in middle panels point to lesions diagnosed as AAHs. The scale bars in the left panels are the same for all panels in each row. (**B**) Statistical analysis of expression differences among the three groups was performed using the Kruskal-Wallis test. Boxes represent 25%–75% ranges and whiskers constitute maxima and minima. Solid horizontal lines represent median values.

We next determined *TBX2* subfamily expression in premalignant precursors of lung cancer. We probed *TBX2* subfamily expression in ongoing transcriptome sequencing efforts to profile global expression changes in normal lung tissues, AAHs and early-stage LUADs acquired from 17 patients. Tissue sections from the samples were histopathologically evaluated (Figure 3A) to confirm normal, premalignant and malignant diagnosis. Transcriptome sequencing analysis (see Materials and Methods) in this cohort revealed significant down-regulation of all four *TBX* genes in AAHs and LUADs relative to normal lung tissues (all *P* < 0.0001 of the Kruskal–Wallis test; Figure 3B). Of note, this observed down-regulation was also progressive with expression of the genes decreased in AAHs and further suppressed and lowest in the LUADs (Figure 3B). These findings suggest that down-regulation of the *TBX2* subfamily is possibly implicated in the development of premalignant precursors (i.e., AAHs) and in the progression of these lesions to malignant LUADs.

### Suppressed expression of the *TBX2* subfamily in the cancerization field in the normal-appearing airway

**Figure 4:**
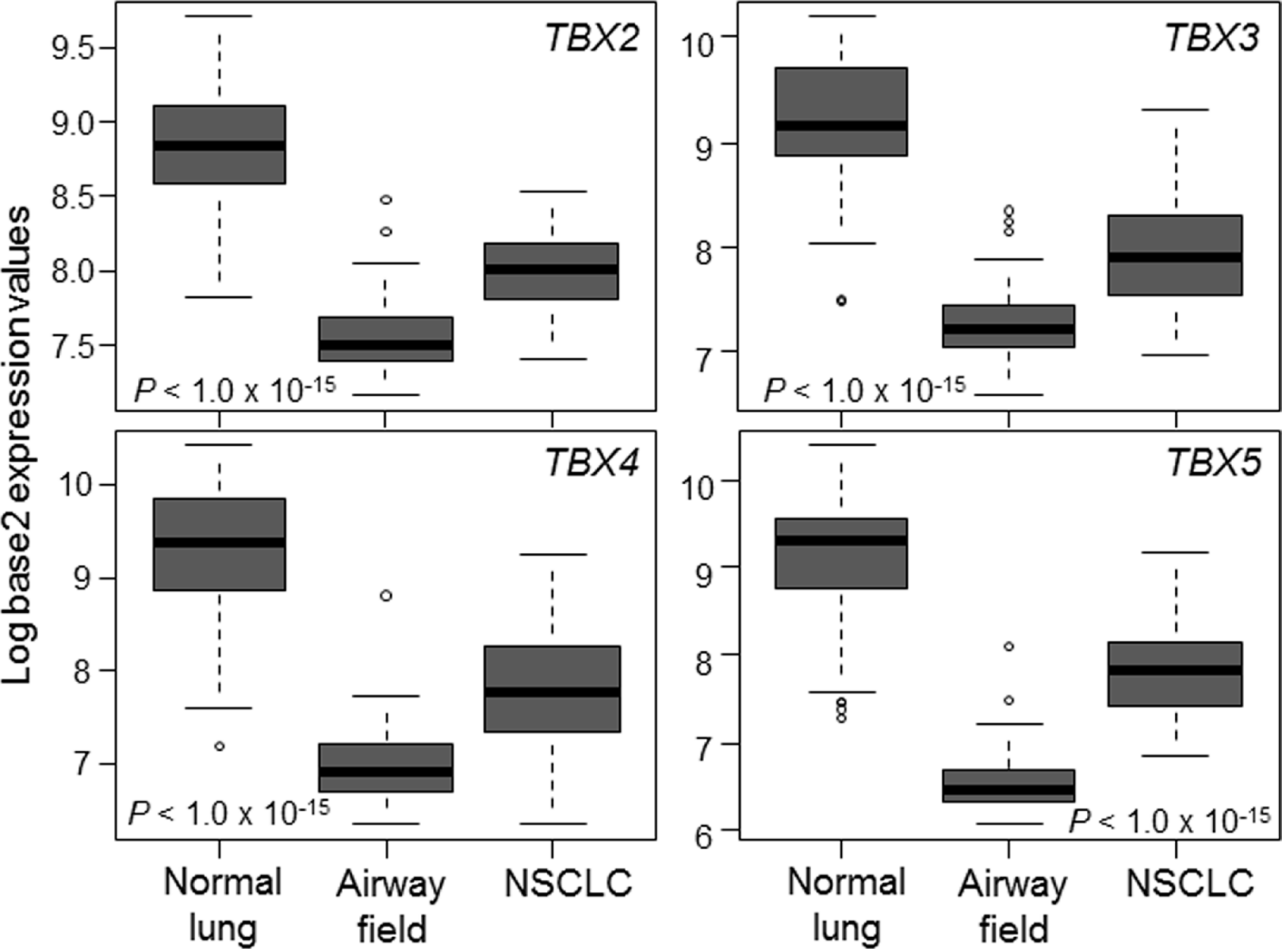
Suppressed expression of the *TBX2* subfamily in the normal-appearing airway cancerization field Expression levels of the *TBX2* subfamily were interrogated in a normalized dataset we previously reported [27] and that is comprised of matched NSCLCs, multiple adjacent cytologically-normal small airway brushings and distant normal lung tissues (see Materials and Methods section). Differences in expression among the three groups were statistically analyzed using the Kruskal-Wallis test. Boxes represent 25%–75% expression ranges and solid horizontal lines represent median values.

Earlier work revealed that there are molecular changes that are shared between adjacent normal-appearing airway epithelial cells and lung tumors themselves, a phenomenon, referred to as airway cancerization field [10]. We statistically interrogated the expression levels in a dataset we recently reported and comprised of matched NSCLCs, multiple adjacent normal-appearing airway brushings and distant normal lung tissues from 20 patients [27]. This analysis revealed that all four members of the *TBX2* subfamily were markedly down-regulated in both NSCLCs and the adjacent airway cancerization field relative to distant normal lung tissues (all *P* < 10^−15^ of the Kruskal–Wallis test; Figure 4).

**Figure 5:**
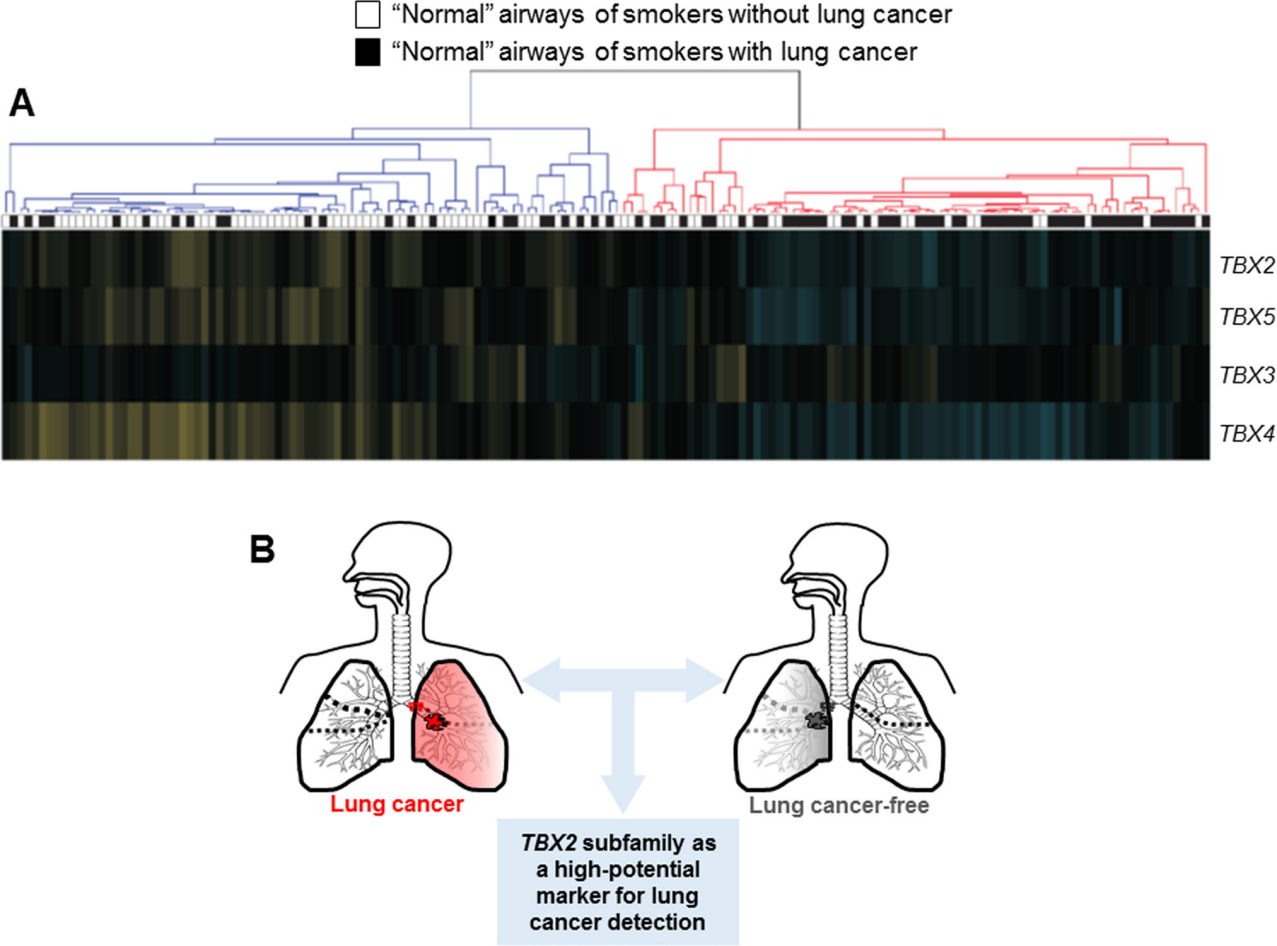
Suppression of the *TBX2* subfamily in the airway field of injury in smokers (**A**) *TBX2* subfamily expression levels were analyzed in the dataset by Spira and colleagues [28] comprised of uninvolved mainstem bronchi from 164 suspect smokers with and without lung cancer. Following re-normalization (see Materials and Methods) section, the samples were clustered based on *TBX2* subfamily expression (blue; down-regulated, yellow; up-regulated compared to the median). Statistical analysis of differences in the numbers of smokers with and without lung cancer between the two clusters was performed using the Fisher's exact test. (**B**) Schematic illustration depicting that the *TBX2* subfamily may serve as a high-potential bronchial four-gene classifier for early detection of lung cancer in smokers.

Previous studies have demonstrated that cigarette smoking induces genome-wide expression changes in the airway suggestive of a “field of injury” [8]. Moreover, this airway field of injury is thought comprise markers for early detection of the disease [8, 10, 27–29]. We studied expression of *TBX2* subfamily in the airway field of injury of smokers with lung cancer contrasting patterns in the field of cancer-free smokers using the dataset by Spira *et al*. comprised of 164 suspect smokers [28]. Expression of the *TBX2* subfamily was reduced in uninvolved mainstem bronchi of smokers with lung cancer relative to cancer-free smokers. Following hierarchical clustering, reduced expression of *TBX2* subfamily in minimally invasive and relatively readily accessible normal large airways was found to be a significantly (*P* < 10^−5^ of the Fisher's exact test) associated with lung cancer in the suspect high-risk smokers (Figure 5). These findings suggest that suppressed expression of the *TBX2* subfamily likely occurs very early on in NSCLC pathogenesis and may serve as a viable biomarker for early detection of lung cancer among suspect smokers with indeterminate nodules.

## DISCUSSION

We interrogated the expression of the *TBX2* subfamily of transcription factors in early phases of human NSCLC pathogenesis. We first found that the four members of the subfamily were preferentially up-regulated in human normal lung tissues as well as consistently suppressed in human lung NSCLCs concomitant with aberrant modulation of various downstream *TBX* target canonical cancer-associated markers. We revealed by transcriptomic analysis that the *TBX* genes were significantly suppressed in premalignant lung lesions (AAHs) and in the cancerization field in the tumor-adjacent normal-appearing airway relative to uninvolved normal lung. Lastly, we found that suppressed expression of the *TBX2* subfamily in cytologically-normal mainstem bronchi significantly distinguished smokers with lung cancer from cancer-free smokers. Our findings demonstrate that suppression of the *TBX2* subfamily occurs early on in human NSCLC pathogenesis and exhibits diagnostic properties of a candidate high-potential biomarker for early detection of lung cancer in high-risk smokers.

We demonstrated concomitant aberration of cancer-associated downstream targets of the *TBX2* subfamily. We surveyed a *TBX5*-mediated gene network and found that it comprised up-regulated expression of baculoviral IAP repeat containing 5 (*BIRC5*), a member of the inhibitor of apoptosis (IAP) family that plays crucial anti-apoptotic roles in cancer [30]. We also found that the modulated *TBX2*-gene neighborhood in LUADs comprised concomitant up-regulation of numerous canonical oncogenes. These included, among others, known promoters of cell cycle progression, carcinogenesis and inhibitors of apoptosis such as aurora kinase A *(AURKA*), budding uninhibited by benzimidazoles *(BUB1*) and cyclin-dependent kinase 1 (*CDK1*) [31, 32]. Our findings are in line with a previous integrative profiling study of a small set (*n* = 6) of East Asian non-smoker LUADs that computationally predicted inhibition of the *TBX2* subfamily member *TBX5* in LUADs relative to normal lungs [33]. Our findings support the supposition that the *TBX2* subfamily may function as a tumor suppressor in human NSCLC.

We also found, by transcriptome sequencing, that suppression of the *TBX2* subfamily occurs in AAHs, lesions that represent the only known precursors for human LUAD and the biology of which is still poorly understood. Among the few molecular aberrations that were reported in AAH lesions are loss of heterozygosity (LOH) in chromosomal regions 3p, 9p, 9q, 17q and 17p [7]. It is noteworthy that *TBX2* and *TBX4* map to 17q23 [34–36] suggesting that reduced expression of both AAHs and LUADs may simply be a surrogate of 17q23 LOH. Future studies are warranted to functionally probe the effect of *TBX2* subfamily expression on the malignant phenotype of NSCLC. Nonetheless, it is intriguing to speculate that suppressed expression of the *TBX2* subfamily may serve as a putative marker for progression of premalignant lesions to malignant lung tumors. It is worthwhile to note that *Tbx2* subfamily members in the mouse were shown to be highly expressed, relative to airway epithelium, in the lung mesenchyme [17]. It is possible that suppression of *TBX2* subfamily expression in premalignant and malignant lung lesions may be due to an expansion of *TBX*-negative lung epithelial cells during lung oncogenesis. Yet, our findings insinuate that *TBX2* subfamily is adequately expressed in human airway epithelium and normal lung parenchyma. Our supposition is supported by the following findings: 1) expression levels of all four members of the *TBX2* subfamily were highest in human normal lung tissue among many (> 7,000) pan-normal human tissues form various organs; 2) the *TBX2* subfamily was markedly and significantly higher in normal lung parenchyma relative to premalignant lesions as well as to NSCLCs from various datasets and, notably, 3) the *TBX2* subfamily was even highly expressed in airway/bronchial epithelial brushings (comprising greater than 98% airway epithelial cells from the mainstem bronchus; [28]) obtained from smokers without lung cancer relative to brushings from smokers diagnosed with lung malignancy. Further, our transcriptome sequencing findings are corroborated by the recent report by Ma and colleagues demonstrating suppressed *TBX5* expression in NSCLC [37]. Forthcoming work is warranted to validate the expression of the *TBX2* subfamily in lung preneoplasia using orthogonal measures (e.g., immunohistochemistry).

We recently defined the transcriptomic architecture of the cancerization field in the normal-appearing airway adjacent to tumors [27]. Here, we demonstrate that all four members of the *TBX2* subfamily are consistently and significantly suppressed in the adjacent normal-appearing airway cancerization field in early-stage NSCLC patients. Our findings suggest that the *TBX2* subfamily is suppressed in early (“normal”) phases in NSCLC pathogenesis. Of note, it is reasonable to speculate that the *TBX2* subfamily may function to prevent loss of differentiation of airway cells owing to a) the known roles of the subfamily in normal lung cell differentiation and organogenesis [18, 19] and b) our present findings on significant suppression of the genes in the normal-appearing airway cancerization field. Of note, earlier studies demonstrated that smoking perpetuates a widespread “field of injury” in the airway that includes genome-wide expression changes thought to be significant for early detection [8, 38]. We analyzed expression of the *TBX2* subfamily in a dataset comprised of cytologically-normal epithelial brushings of uninvolved mainstem bronchi obtained from suspect smokers during bronchoscopy [28]. We found that not only the subfamily was down-regulated in normal bronchi of smokers with lung cancer compared to cancer-free smokers but also that expression of the genes was indicative of lung cancer status. Endoscopic bronchoscopy is sometimes non-diagnostic in the clinical setting particularly among smokers with intermediate-risk [29]. Recent studies have derived bronchial-gene classifiers that improve the specificity of bronchoscopy among patients with an intermediate pretest probability of cancer. Our findings warrant future studies to validate the diagnostic capacity of the putative *TBX2* four-gene classifier in additional cohorts and in combination with bronchoscopy.

Earlier reports have suggested that expression levels of *TBX2* are elevated in NSCLC [39, 40]. The reports by Hu et al. [39] and Zhang et al. [40] interrogated TBX2 protein by immunohistochemistry in NSCLCs alone (tissue microarrays) and suggested that tumors with relatively high expression of the protein (based on percent positivity within the tumors themselves) exhibited comparatively poor prognosis [39, 40]. The same reports studied expression levels of the *TBX2* gene (e.g. by quantitative real-time PCR) in NSCLCs and paired normal lung tissues in a smaller subset of NSCLC cases (*n* = 40 to 50) [39, 40] demonstrating that the gene is elevated in the tumors. These earlier studies [39–41], albeit few, point to putative oncogenic roles and properties for the *TBX2* gene in human NSCLC. In contrast, our findings, collectively, revealed that the *TBX2* gene is markedly suppressed in NSCLC relative to normal lung from four publicly available expression datasets [23–26] (Supplementary Figure 3). Of note, we replicated these findings in six additional independent cohorts [42–47] of NSCLCs and normal lung tissues (Supplementary Figure 4) — all consistently showing significantly suppressed expression of *TBX2* in NSCLC. Using ten cohorts of over 750 NSCLCs, that had originally employed different platforms (e.g. Affymetrix and Illumina), our study strongly points to suppressed levels of *TBX2* mRNA in the lung malignancy. A comparison and contrast of our study's design and conclusions to the aforementioned earlier reports by Hu et al. and Zhang et al. [39, 40] may explain this stark discrepancy. For one, we studied all members of the *TBX2* subfamily demonstrating that all four members are down-regulated in different phases in the pathogenesis of human NSCLC. We also demonstrated that the four members of the *TBX2* subfamily are highest in normal lung tissues relative to all other normal tissues from other organs using transcriptome sequencing data from over 7,000 specimens thus clearly showing relatively high expression of the factors in normal lung. We interrogated complimentary datasets and cohorts that utilized different technologies (arrays and next-generation sequencing) and comprised different phases in the pathogenesis of NSCLC, namely premalignant lung lesions and cytologically-normal airway epithelia in the cancerization field adjacent to tumors — all pointing to suppressed expression of the *TBX2* subfamily in NSCLC. Further, we successfully extended our findings to early detection of lung cancer by also demonstrating reduced expression of the subfamily in airways of smokers with lung cancer relative to those without the malignancy (from an independent cohort) thus providing a non-linear proof-of-principle validation of reduced expression of *TBX2* subfamily in early-stage lung cancer. Our findings on suppressed expression of *TBX2* subfamily in NSCLC are also in congruence with various recent reports implicating tumor suppressive properties for members of the *TBX2* subfamily. While the report by Wu et al. [41] showed that *TBX3* is up-regulated in NSCLC, the same study demonstrated that a fraction of NSCLCs show reduced expression of the gene. Low expression of *TBX4* gene was reported to be associated with worse prognosis among patients with stage II pancreatic ductal adenocarcinoma [48]. Ectopic over-expression of TBX5 protein was demonstrated to inhibit colon cancer cell proliferation and induce apoptosis thus functioning as a putative tumor suppressor [49]. A recent study by Ma and colleagues [37] demonstrated that expression of *TBX5*, is suppressed in NSCLC and over-expression of the gene inhibits proliferation and invasion in NSCLC cell lines. Also, the report by Du et al. [33], as mentioned earlier, computationally demonstrated, by DNA methylation and microRNA profiling analyses, inhibition of the *TBX5* factor in human LUADs relative to normal tissues. Our extended findings may be, in part, due to the relatively more comprehensive nature of our study that employed a larger cohort of profiled NSCLCs, different profiling technologies and different phases (normal-appearing and premalignant) of human NSCLC.

In conclusion, we demonstrate that the *TBX2* subfamily is markedly decreased in human premalignant and malignant lung lesions compared to normal lung. We also report that the *TBX2* subfamily is suppressed in the normal-appearing airway cancerization field in NSCLC. Further, we found that suppressed expression of the *TBX2* subfamily in normal and minimally invasive sites in the lung is diagnostic of lung cancer. Our study provides evidence that the *TBX2* subfamily may serve as a high-potential bronchial four-gene biomarker for early detection of lung cancer in suspect smokers.

## MATERIALS AND METHODS

### *In silico* analysis of *TBX2* subfamily expression in normal and malignant lung tissues

Levels of *TBX2, TBX3, TBX4* and *TBX5* mRNAs were assessed in expression data of > 7,000 pan-normal tissues from the Genotype-Tissue Expression (GTEx) project version V6p [22]. *TBX2* subfamily expression was analyzed in our previously reported dataset of LUADs (*n* = 80) and normal lung tissues (*n* = 30) [23]. Hierarchical clustering of the LUADs and normal lung tissues was performed in R. Expression correlation analysis of *TBX2* subfamily was performed using Pearson's correlation. Topological network analysis of gene targets downstream of *TBX2* subfamily was performed using Ingenuity Pathways Analysis (IPA) [23]. Expression levels of the *TBX2* subfamily members were also statistically analyzed, using the Wilcoxon rank sum test, in three independent publicly available array datasets [24–26].

### Cohort of atypical adenomatous hyperplasias

Matched normal lung tissues, atypical adenomatous hyperplasias (AAHs) and early-stage (stages I and II) LUADs were studied by transcriptome sequencing. Cases with available AAHs were included in the analysis (*n* = 17 patients). Formalin-fixed paraffin embedded (FFPE) specimens were obtained from the Aichi Cancer Center (Nagoya, Japan) and Nagasaki University (Nagasaki, Japan) and were approved for study by institutional review boards (IRBs). LUAD staging and pathological assessment was determined in the manner previously reported by Travis and colleagues [50]. Histopathology assessment of AAHs and LUADs was performed by analysis of H&E stained alternating slides (from 5 micron sections) with sections in between (10 micron) preserved for RNA isolation.

### Transcriptome sequencing analysis

Five to 15 sections/slides per specimen were deparaffinized prior to scraping of normal tissues and lesions with 25-gauge needles under a stereomicroscope. Tissue fragments were then collected in lysis buffer PKD (Qiagen). Total RNA was isolated using the AllPrep DNA/RNA FFPE kit from Qiagen following the manufacturer's protocol. Concentrations were measured on a NanoDrop 1000 (Thermo) and RNA integrity numbers (RINs) were obtained on the 2100 Bioanalyzer (Agilent Technologies) using the RNA 6000 Nano or Pico kit.

RNA (30 ng) were reverse-transcribed and barcoded cDNA libraries were generated using the Ion AmpliSeq Transcriptome Human Gene Expression Kit (Thermo Fisher Scientific) adhering to the manufacturer's protocol for FFPE samples. Library concentrations were determined using the Ion Library TaqMan Quantitation Kit. Specimens from two cases were processed together in one sequencing chip. Template reactions were carried out using the Ion PI Hi-Q OT2 200 Kit and then loaded onto Ion PI chips v3 using the Ion PI Hi-Q Sequencing 200 Kit based (Thermo Fisher Scientific). All samples were sequenced on an Ion Proton sequencer. The Ion Torrent Suite 5.0 was used for assessing quality of the libraries and sequencing runs. Base calling results for each sample were aligned to a reference file (hg19_ampliseq_transcriptome_ercc_v1.fasta) standard to the Ion Torrent Suite software. The aligned files were then transferred over to the Partek flow software and transcriptomes from the mapped .bam files were quantified using a modified version of the expectation-maximization (E/M) algorithm as described previously [51]. Resultant transcript counts were log (base 2) transformed. Expression levels of the *TBX2* subfamily were extracted, analyzed by Kruskal–Wallis test among the different groups and plotted using R.

### Analysis of *TBX2* subfamily expression in the cancerization field in the normal-appearing airway

*TBX2* subfamily expression levels were analyzed in matched early-stage NSCLCs, brushings from multiple tumor-adjacent small airways and distant normal tissues from 20 patients, from a dataset we previously reported [27], using the Kruskal–Wallis test and R. Expression levels of the subfamily were assessed in the dataset by Spira *et al*. [28] comprised of normal epithelial brushings from the uninvolved mainstem bronchus of 164 lifetime smokers (78 with lung cancer and 86 cancer-free) with suspicion of lung cancer. The CEL files corresponding to the training and test from the Spira *et al*. study were re-normalized to produce gene-level expression values by robust multiarray analysis in the Bioconductor software suite [52]. A linear model was used to adjust the expression estimates for the mean *Z* score quality metric. Differential gene expression between airways from healthy smokers and those with lung cancer was assessed using two-tailed Student's *t* test. Differences in the number of smokers with lung cancer and those without the malignancy between clusters derived based on *TBX2* subfamily expression were statistically assessed using Fisher's exact test. Similar analysis of the *TBX2* subfamily was performed in an additional dataset GSE66499 [29] of normal large airways from 680 suspect smokers.

## SUPPLEMENTARY MATERIALS AND FIGURES



## References

[1] Siegel RL, Miller KD, Jemal A (2016). Cancer statistics, 2016. CA Cancer J Clin.

[2] Villalobos P, Wistuba II (2017). Lung Cancer Biomarkers. Hematol Oncol Clin North Am.

[3] Herbst RS, Heymach JV, Lippman SM (2008). Lung cancer. N Engl J Med.

[4] Gold KA, Kim ES, Lee JJ, Wistuba II, Farhangfar CJ, Hong WK (2011). The BATTLE to personalize lung cancer prevention through reverse migration. Cancer Prev Res (Phila).

[5] Wistuba II, Gelovani JG, Jacoby JJ, Davis SE, Herbst RS (2011). Methodological and practical challenges for personalized cancer therapies. Nat Rev Clin Oncol.

[6] Aberle DR, Adams AM, Berg CD, Black WC, Clapp JD, Fagerstrom RM, Gareen IF, Gatsonis C, Marcus PM, Sicks JD, National Lung Screening Trial Research T (2011). Reduced lung-cancer mortality with low-dose computed tomographic screening. N Engl J Med.

[7] Kadara H, Kabbout M, Wistuba II (2012). Pulmonary adenocarcinoma: a renewed entity in 2011. Respirology.

[8] Steiling K, Ryan J, Brody JS, Spira A (2008). The field of tissue injury in the lung and airway. Cancer Prev Res (Phila).

[9] Gower AC, Steiling K, Brothers JF, Lenburg ME, Spira A (2011). Transcriptomic studies of the airway field of injury associated with smoking-related lung disease. Proc Am Thorac Soc.

[10] Kadara H, Wistuba II (2012). Field cancerization in non-small cell lung cancer: implications in disease pathogenesis. Proc Am Thorac Soc.

[11] Naiche LA, Harrelson Z, Kelly RG, Papaioannou VE (2005). T-box genes in vertebrate development. Annu Rev Genet.

[12] Hariri F, Nemer M, Nemer G (2012). T-box factors: insights into the evolutionary emergence of the complex heart. Ann Med.

[13] Papaioannou VE (2014). The T-box gene family: emerging roles in development, stem cells and cancer. Development.

[14] Packham EA, Brook JD (2003). T-box genes in human disorders. Hum Mol Genet.

[15] Horton AC, Mahadevan NR, Minguillon C, Osoegawa K, Rokhsar DS, Ruvinsky I, de Jong PJ, Logan MP, Gibson-Brown JJ (2008). Conservation of linkage and evolution of developmental function within the Tbx2/3/4/5 subfamily of T-box genes: implications for the origin of vertebrate limbs. Dev Genes Evol.

[16] Gibson-Brown JJ, I Agulnik S, Silver LM, Papaioannou VE (1998). Expression of T-box genes Tbx2-Tbx5 during chick organogenesis. Mech Dev.

[17] Ludtke TH, Farin HF, Rudat C, Schuster-Gossler K, Petry M, Barnett P, Christoffels VM, Kispert A (2013). Tbx2 controls lung growth by direct repression of the cell cycle inhibitor genes Cdkn1a and Cdkn1b. PLoS Genet.

[18] Ludtke TH, Rudat C, Wojahn I, Weiss AC, Kleppa MJ, Kurz J, Farin HF, Moon A, Christoffels VM, Kispert A (2016). Tbx2 and Tbx3 Act Downstream of Shh to Maintain Canonical Wnt Signaling during Branching Morphogenesis of the Murine Lung. Dev Cell.

[19] Arora R, Metzger RJ, Papaioannou VE (2012). Multiple roles and interactions of Tbx4 and Tbx5 in development of the respiratory system. PLoS Genet.

[20] Cebra-Thomas JA, Bromer J, Gardner R, Lam GK, Sheipe H, Gilbert SF (2003). T-box gene products are required for mesenchymal induction of epithelial branching in the embryonic mouse lung. Dev Dyn.

[21] Chapman DL, Garvey N, Hancock S, Alexiou M, Agulnik SI, Gibson-Brown JJ, Cebra-Thomas J, Bollag RJ, Silver LM, Papaioannou VE (1996). Expression of the T-box family genes, Tbx1-Tbx5, during early mouse development. Dev Dyn.

[22] Consortium GT (2013). The Genotype-Tissue Expression (GTEx) project. Nat Genet.

[23] Kabbout M, Garcia MM, Fujimoto J, Liu DD, Woods D, Chow CW, Mendoza G, Momin AA, James BP, Solis L, Behrens C, Lee JJ, Wistuba II (2013). ETS2 mediated tumor suppressive function and MET oncogene inhibition in human non-small cell lung cancer. Clin Cancer Res.

[24] Okayama H, Kohno T, Ishii Y, Shimada Y, Shiraishi K, Iwakawa R, Furuta K, Tsuta K, Shibata T, Yamamoto S, Watanabe S, Sakamoto H, Kumamoto K (2012). Identification of genes upregulated in ALK-positive and EGFR/KRAS/ALK-negative lung adenocarcinomas. Cancer Res.

[25] Hou J, Aerts J, den Hamer B, van Ijcken W, den Bakker M, Riegman P, van der Leest C, van der Spek P, Foekens JA, Hoogsteden HC, Grosveld F, Philipsen S (2010). Gene expression-based classification of non-small cell lung carcinomas and survival prediction. PLoS One.

[26] Wei TY, Juan CC, Hisa JY, Su LJ, Lee YC, Chou HY, Chen JM, Wu YC, Chiu SC, Hsu CP, Liu KL, Yu CT (2012). Protein arginine methyltransferase 5 is a potential oncoprotein that upregulates G1 cyclins/cyclin-dependent kinases and the phosphoinositide 3-kinase/AKT signaling cascade. Cancer Sci.

[27] Kadara H, Fujimoto J, Yoo SY, Maki Y, Gower AC, Kabbout M, Garcia MM, Chow CW, Chu Z, Mendoza G, Shen L, Kalhor N, Hong WK (2014). Transcriptomic architecture of the adjacent airway field cancerization in non-small cell lung cancer. J Natl Cancer Inst.

[28] Spira A, Beane JE, Shah V, Steiling K, Liu G, Schembri F, Gilman S, Dumas YM, Calner P, Sebastiani P, Sridhar S, Beamis J, Lamb C (2007). Airway epithelial gene expression in the diagnostic evaluation of smokers with suspect lung cancer. Nat Med.

[29] Silvestri GA, Vachani A, Whitney D, Elashoff M, Porta Smith K, Ferguson JS, Parsons E, Mitra N, Brody J, Lenburg ME, Spira A, Team AS (2015). A Bronchial Genomic Classifier for the Diagnostic Evaluation of Lung Cancer. N Engl J Med.

[30] Silke J, Vaux DL (2001). Two kinds of BIR-containing protein - inhibitors of apoptosis, or required for mitosis. J Cell Sci.

[31] Dos Santos EO, Carneiro-Lobo TC, Aoki MN, Levantini E, Basseres DS (2016). Aurora kinase targeting in lung cancer reduces KRAS-induced transformation. Mol Cancer.

[32] McKay JA, Douglas JJ, Ross VG, Curran S, Murray GI, Cassidy J, McLeod HL (2000). Cyclin D1 protein expression and gene polymorphism in colorectal cancer. Aberdeen Colorectal Initiative. Int J Cancer.

[33] Du J, Zhang L (2015). Integrated analysis of DNA methylation and microRNA regulation of the lung adenocarcinoma transcriptome. Oncol Rep.

[34] Lu W, Bacino CA, Richards BS, Alvarez C, VanderMeer JE, Vella M, Ahituv N, Sikka N, Dietz FR, Blanton SH, Hecht JT (2012). Studies of TBX4 and chromosome 17q23.1q23.2: an uncommon cause of nonsyndromic clubfoot. Am J Med Genet A.

[35] Wang B, Lindley LE, Fernandez-Vega V, Rieger ME, Sims AH, Briegel KJ (2012). The T box transcription factor TBX2 promotes epithelial-mesenchymal transition and invasion of normal and malignant breast epithelial cells. PLoS One.

[36] Dimova I, Orsetti B, Negre V, Rouge C, Ursule L, Lasorsa L, Dimitrov R, Doganov N, Toncheva D, Theillet C (2009). Genomic markers for ovarian cancer at chromosomes 1, 8 and 17 revealed by array CGH analysis. Tumori.

[37] Ma R, Yang Y, Tu Q, Hu K (2017). Overexpression of T-box Transcription Factor 5 (TBX5) Inhibits Proliferation and Invasion in. Non-Small Cell Lung Carcinoma Cells. Oncol Res.

[38] Kadara H, Scheet P, Wistuba II, Spira AE (2016). Early Events in the Molecular Pathogenesis of Lung Cancer. Cancer Prev Res (Phila).

[39] Hu B, Mu HP, Zhang YQ, Su CY, Song JT, Meng C, Liu DX (2014). Prognostic significance of TBX2 expression in non-small cell lung cancer. J Mol Histol.

[40] Zhang Z, Guo Y (2014). High TBX2 expression predicts poor prognosis in non-small cell lung cancer. Neoplasma.

[41] Wu Y, Feng J, Hu W, Zhang Y (2017). T-box 3 overexpression is associated with poor prognosis of non-small cell lung cancer. Oncol Lett.

[42] Beer DG, Kardia SL, Huang CC, Giordano TJ, Levin AM, Misek DE, Lin L, Chen G, Gharib TG, Thomas DG, Lizyness ML, Kuick R, Hayasaka S (2002). Gene-expression profiles predict survival of patients with lung adenocarcinoma. Nat Med.

[43] Bhattacharjee A, Richards WG, Staunton J, Li C, Monti S, Vasa P, Ladd C, Beheshti J, Bueno R, Gillette M, Loda M, Weber G, Mark EJ (2001). Classification of human lung carcinomas by mRNA expression profiling reveals distinct adenocarcinoma subclasses. Proc Natl Acad Sci USA.

[44] Landi MT, Dracheva T, Rotunno M, Figueroa JD, Liu H, Dasgupta A, Mann FE, Fukuoka J, Hames M, Bergen AW, Murphy SE, Yang P, Pesatori AC (2008). Gene expression signature of cigarette smoking and its role in lung adenocarcinoma development and survival. PLoS One.

[45] Selamat SA, Chung BS, Girard L, Zhang W, Zhang Y, Campan M, Siegmund KD, Koss MN, Hagen JA, Lam WL, Lam S, Gazdar AF, Laird-Offringa IA (2012). Genome-scale analysis of DNA methylation in lung adenocarcinoma and integration with mRNA expression. Genome Res.

[46] Stearman RS, Dwyer-Nield L, Zerbe L, Blaine SA, Chan Z, Bunn PA, Johnson GL, Hirsch FR, Merrick DT, Franklin WA, Baron AE, Keith RL, Nemenoff RA (2005). Analysis of orthologous gene expression between human pulmonary adenocarcinoma and a carcinogen-induced murine model. Am J Pathol.

[47] Su LJ, Chang CW, Wu YC, Chen KC, Lin CJ, Liang SC, Lin CH, Whang-Peng J, Hsu SL, Chen CH, Huang CY (2007). Selection of DDX5 as a novel internal control for Q-RT-PCR from microarray data using a block bootstrap re-sampling scheme. BMC Genomics.

[48] Zong M, Meng M, Li L (2011). Low expression of TBX4 predicts poor prognosis in patients with stage II pancreatic ductal adenocarcinoma. Int J Mol Sci.

[49] Yu J, Ma X, Cheung KF, Li X, Tian L, Wang S, Wu CW, Wu WK, He M, Wang M, Ng SS, Sung JJ (2010). Epigenetic inactivation of T-box transcription factor 5, a novel tumor suppressor gene, is associated with colon cancer. Oncogene.

[50] Travis WD, Brambilla E, Nicholson AG, Yatabe Y, Austin JH, Beasley MB, Chirieac LR, Dacic S, Duhig E, Flieder DB, Geisinger K, Hirsch FR, Ishikawa Y (2015). The 2015 World Health Organization Classification of Lung Tumors: Impact of Genetic, Clinical and Radiologic Advances Since the 2004 Classification. J Thorac Oncol.

[51] Li B, Ruotti V, Stewart RM, Thomson JA, Dewey CN (2010). RNA-Seq gene expression estimation with read mapping uncertainty. Bioinformatics.

[52] Gautier L, Cope L, Bolstad BM, Irizarry RA (2004). affy—analysis of Affymetrix GeneChip data at the probe level. Bioinformatics.

