# Supplementary material for: TBX2 subfamily suppression in lung cancer pathogenesis: a high-potential marker for early detection
Source: Oncotarget. 2017 Aug 4;8(40):68230–41. doi: 10.18632/oncotarget.19938 (PMC5620251; doi:10.18632/oncotarget.19938)
Supplement: Supplementary file 1 [file oncotarget-08-68230-s001.pdf]

TBX2 subfamily suppression in lung cancer pathogenesis: a high-potential marker for early detection

SUPPLEMENTARY MATERIALS

SUPPLEMENTARY REFERENCES

1. Kabbout M, Garcia MM, Fujimoto J, Liu DD, Woods D, Chow CW, Mendoza G, Momin AA, James BP, Solis L, Behrens C, Lee JJ, Wistuba II, et al. ETS2 mediated tumor suppressive function and MET oncogene inhibition in human non-small cell lung cancer. Clin Cancer Res. 2013; 19:3383–3395.
2. Okayama H, Kohno T, Ishii Y, Shimada Y, Shiraishi K, Iwakawa R, Furuta K, Tsuta K, Shibata T, Yamamoto S, Watanabe S, Sakamoto H, Kumamoto K, et al. Identification of genes upregulated in ALK-positive and EGFR/KRAS/ALK-negative lung adenocarcinomas. Cancer Res. 2012; 72:100–111.
3. Hou J, Aerts J, den Hamer B, van Ijcken W, den Bakker M, Riegman P, van der Leest C, van der Spek P, Foekens JA, Hoogsteden HC, Grosveld F, Philipsen S. Gene expression-based classification of non-small cell lung carcinomas and survival prediction. PloS one. 2010; 5:e10312.
4. Wei TY, Juan CC, Hisa JY, Su LJ, Lee YC, Chou HY, Chen JM, Wu YC, Chiu SC, Hsu CP, Liu KL, Yu CT. Protein arginine methyltransferase 5 is a potential oncoprotein that upregulates G1 cyclins/cyclin-dependent kinases and the phosphoinositide 3-kinase/AKT signaling cascade. Cancer science. 2012; 103:1640–1650.
5. Beer DG, Kardia SL, Huang CC, Giordano TJ, Levin AM, Misek DE, Lin L, Chen G, Gharib TG, Thomas DG, Lizyness ML, Kuick R, Hayasaka S, et al. Gene-expression profiles predict survival of patients with lung adenocarcinoma. Nat Med. 2002; 8:816–824.
6. Bhattacharjee A, Richards WG, Staunton J, Li C, Monti S, Vasa P, Ladd C, Beheshti J, Bueno R, Gillette M, Loda M, Weber G, Mark EJ, et al. Classification of human lung carcinomas by mRNA expression profiling reveals distinct adenocarcinoma subclasses. Proc Natl Acad Sci USA. 2001; 98:13790–13795.
7. Landi MT, Dracheva T, Rotunno M, Figueroa JD, Liu H, Dasgupta A, Mann FE, Fukuoka J, Hames M, Bergen AW, Murphy SE, Yang P, Pesatori AC, et al. Gene expression signature of cigarette smoking and its role in lung adenocarcinoma development and survival. PloS one. 2008; 3:e1651.
8. Selamat SA, Chung BS, Girard L, Zhang W, Zhang Y, Campan M, Siegmund KD, Koss MN, Hagen JA, Lam WL, Lam S, Gazdar AF, Laird-Offringa IA. Genome-scale analysis of DNA methylation in lung adenocarcinoma and integration with mRNA expression. Genome research. 2012; 22:1197–1211.
9. Stearman RS, Dwyer-Nield L, Zerbe L, Blaine SA, Chan Z, Bunn PA Jr., Johnson GL, Hirsch FR, Merrick DT, Franklin WA, Baron AE, Keith RL, et al. Analysis of orthologous gene expression between human pulmonary adenocarcinoma and a carcinogen-induced murine model. The American journal of pathology. 2005; 167:1763–1775.
10. Su LJ, Chang CW, Wu YC, Chen KC, Lin CJ, Liang SC, Lin CH, Whang-Peng J, Hsu SL, Chen CH, Huang CY. Selection of DDX5 as a novel internal control for Q-RT-PCR from microarray data using a block bootstrap re-sampling scheme. BMC Genomics. 2007; 8:140.

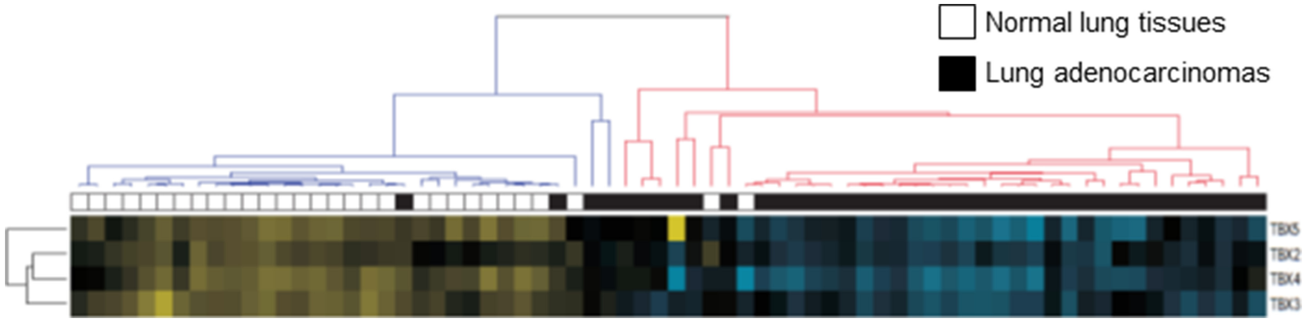

Supplementary Figure 1: Expression of the *TBX2* subfamily distinguishes NSCLCs from normal lung tissues. Expression levels of *TBX2* subfamily were probed in a normalized expression dataset of LUADs and normal lung tissues we previously reported [1]. Samples were then analyzed by hierarchical clustering (in R) based on centered expression of the four members of the *TBX2* subfamily (blue; down-regulated, yellow; up-regulated compared to the median). Statistical analysis in differences in the numbers of NSCLCs and normal lung tissues between the two clusters was performed in R using the Fisher's exact test.

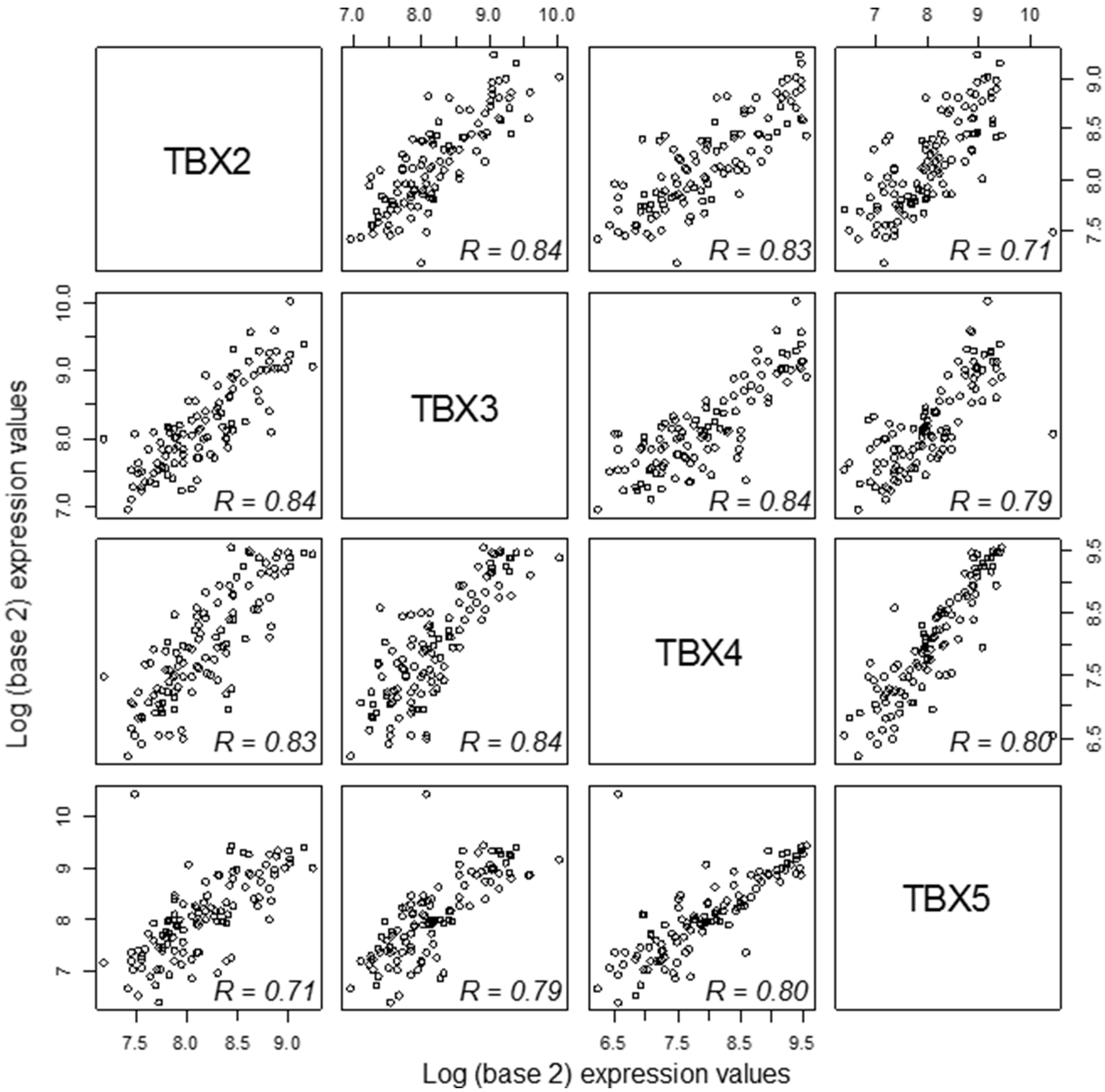

Supplementary Figure 2: Expression levels of members of the *TBX2* subfamily are highly positively correlated in NSCLCs and normal lung tissues. Expression correlation of *TBX2*, *TBX3*, *TBX4* and *TBX5* messenger RNAs (mRNAs) in the LUADs and normal lung tissues (from Figure 2) were statistically analyzed using the Pearson correlation method. Correlation plots were generated in R.

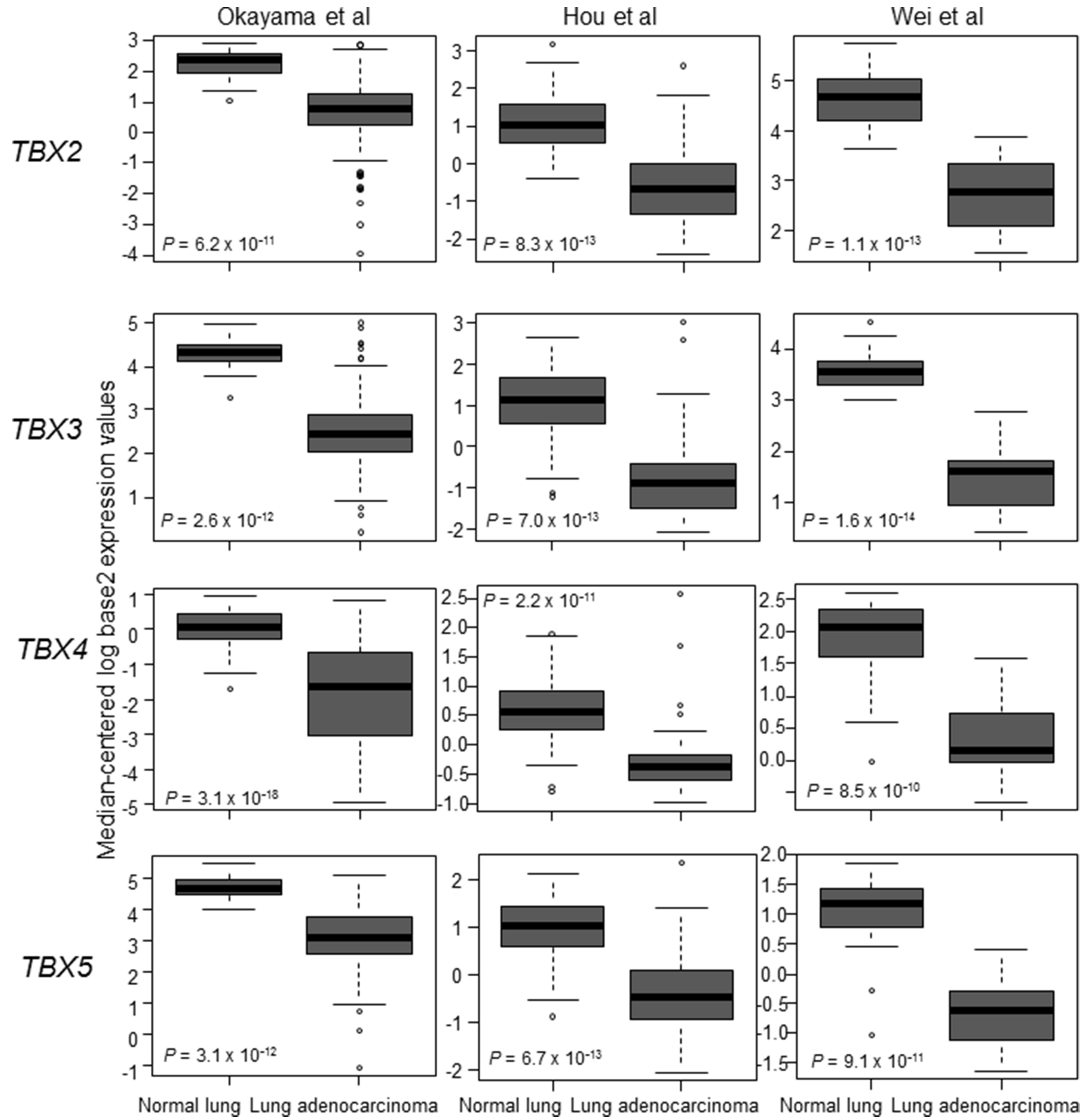

Supplementary Figure 3: The *TBX2* subfamily is widely and commonly suppressed in different cohorts of human NSCLC. Expression levels of *TBX2*, *TBX3*, *TBX4* and *TBX5* were analyzed in three independent publicly available expression datasets of human NSCLCs and normal lung tissues by Okayama et al, Hou et al and Wei and colleagues [2–4]. Differences in expression of the genes between NSCLCs and normal lung tissues were statistically determined using the Wilcoxon rank sum test method in R. Boxes represent 25%–75% expression ranges and whiskers constitute maxima and minima. Solid horizontal lines represent median mRNA expression values of the *TBX* genes.

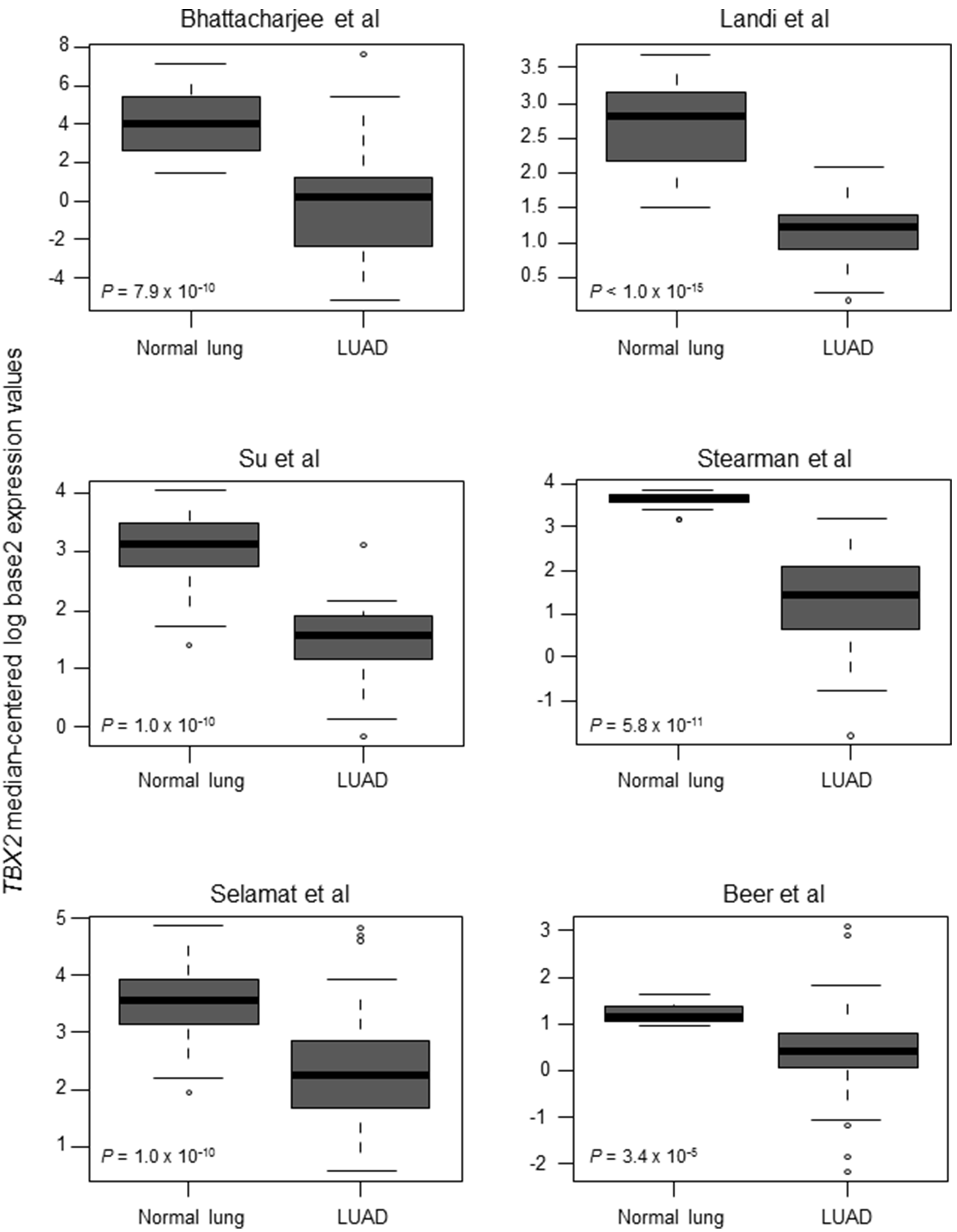

Supplementary Figure 4: Expression levels of the *TBX2* gene are commonly significantly suppressed in human NSCLC. Expression levels of *TBX2* were analyzed in six additional independent publicly available expression datasets of human NSCLCs (LUADs) and normal lung tissues by Bhattacharjee et al, Landi et al, Su et al, Sterman et al, Selamat et al and Beer and colleagues [5–10]. Differences in expression levels of *TBX2* gene between LUADs and normal lung tissues were statistically determined using the Wilcoxon rank sum test method in R. Boxes represent 25%–75% expression ranges and whiskers constitute maxima and minima. Solid horizontal lines represent median mRNA expression values of *TBX2*.
